# Digital Outcome Measurement to Improve Care for Patients With Immune-Mediated Inflammatory Diseases: Protocol for the IMID Registry

**DOI:** 10.2196/43230

**Published:** 2023-03-30

**Authors:** Agnes E M Looijen, Reinier C A van Linschoten, Jan-Dietert Brugma, Dirk Jan Hijnen, Pascal H P de Jong, P Hugo M van der Kuy, Jan A M van Laar, C Janneke van der Woude, Annelieke Pasma

**Affiliations:** 1 Department of Rheumatology Erasmus MC Rotterdam Netherlands; 2 Department of Gastroenterology and Hepatology Erasmus MC Rotterdam Netherlands; 3 Department of Gastroenterology and Hepatology Franciscus Gasthuis & Vlietland Rotterdam Netherlands; 4 Department of Outpatient Pharmacy Erasmus MC Rotterdam Netherlands; 5 Department of Dermatology Erasmus MC Rotterdam Netherlands; 6 Department of Clinical Pharmacy Erasmus MC Rotterdam Netherlands; 7 Departments of Internal Medicine and Immunology Erasmus MC Rotterdam Netherlands

**Keywords:** immune-mediated inflammatory diseases, IMID, value-based health care, patient-reported outcomes, web-based, tool, disease, patient, inflammatory, medicine, dermatology, rheumatology, gastroenterology, immunology, quality of life, mHealth, web-based tool, digital system

## Abstract

**Background:**

Despite enormous clinical improvements, due to better management strategies and the availability of biologicals, immune-mediated inflammatory diseases (IMIDs) still have a significant impact on patients’ lives. To further reduce disease burden, provider- as well as patient-reported outcomes (PROs) should be taken into account during treatment and follow-up. Web-based collection of these outcomes generates valuable repeated measurements, which could be used (1) in daily clinical practice for patient-centered care, including shared decision-making; (2) for research purposes; and (3) as an essential step toward the implementation of value-based health care (VBHC). Our ultimate goal is that our health care delivery system is completely aligned with the principles of VBHC. For aforementioned reasons, we implemented the IMID registry.

**Objective:**

The IMID registry is a digital system for routine outcome measurement that mainly includes PROs to improve care for patients with IMIDs.

**Methods:**

The IMID registry is a longitudinal observational prospective cohort study within the departments of rheumatology, gastroenterology, dermatology, immunology, clinical pharmacy, and outpatient pharmacy of the Erasmus MC, the Netherlands. Patients with the following diseases are eligible for inclusion: inflammatory arthritis, inflammatory bowel disease, atopic dermatitis, psoriasis, uveitis, Behçet disease, sarcoidosis, and systemic vasculitis. Generic and disease-specific (patient-reported) outcomes, including adherence to medication, side effects, quality of life, work productivity, disease damage, and activity, are collected from patients and providers at fixed intervals before and during outpatient clinic visits. Data are collected and visualized through a data capture system, which is linked directly to the patients’ electronic health record, which not only facilitates a more holistic care approach, but also helps with shared decision-making.

**Results:**

The IMID registry is an ongoing cohort with no end date. Inclusion started in April 2018. From start until September 2022, a total of 1417 patients have been included from the participating departments. The mean age at inclusion was 46 (SD 16) years, and 56% of the patient population is female. The average percentage of filled out questionnaires at baseline is 84%, which drops to 72% after 1 year of follow-up. This decline may be due to the fact that the outcomes are not always discussed during the outpatient clinic visit or because the questionnaires were sometimes forgotten to set out. The registry is also used for research purposes and 92% of the patients with IMIDs gave informed consent to use their data for that.

**Conclusions:**

The IMID registry is a web-based digital system that collects provider- and PROs. The collected outcomes are used to improve care for the individual patient with an IMID and facilitate shared decision-making, and they are also used for research purposes. The measurement of these outcomes is an essential step toward the implementation of VBHC.

**International Registered Report Identifier (IRRID):**

DERR1-10.2196/43230

## Introduction

### Background

Immune-mediated inflammatory diseases (IMIDs) represent a group of chronic conditions that share common pathophysiological pathways that cause acute or chronic inflammation [1,2]. Any organ system can be affected, resulting in a broad variety of conditions including inflammatory arthritis (IA), inflammatory bowel disease (IBD), cutaneous inflammatory conditions, and several immunologic disorders [1,2].

Many IMIDs are treated with immunosuppressive medication, such as biologicals and disease-modifying antirheumatic drugs [1-9]. Over the years, the innovation of management approaches and the development of new treatment options have led to significant improvement in clinical outcomes [1]. However, IMIDs still have a large impact on patients’ lives, including lower quality of life, more disability, and productivity loss [10-12]. Moreover, treatment is also often associated with medication-related adverse events [1,13]. Therefore, we should not only focus on clinical outcomes, such as disease activity measures, but also on outcomes that are relevant for the individual patient.

These “patient-reported outcomes” (PROs) cover topics, such as fatigue, pain, treatment satisfaction, and quality of life, without interpretation by a health care provider [14]. They are measured with PRO measures that mostly consist of validated questionnaires. The measurement of PROs in daily practice has several potential benefits as they could (1) improve care for the individual patient, (2) provide real-world data for research purposes, and (3) serve as a valuable part in the shift toward value-based health care (VBHC).

As PROs provide insight in disease burden and its treatment from a patient’s perspective, they could be complementary to conventional clinical measures that are normally used during outpatient clinic visits. The use of PROs in daily practice helps both the physician and patient in preparing the consultation, but also facilitates the prioritization of topics that need to be addressed, resulting in a more efficient and problem-focused consultation [15]. The health care provider may also have the opportunity to tailor the treatment to the patients’ needs and recognize problems that might otherwise be overlooked [15]. Moreover, visualizing PROs over time makes it easier for both patient and physician to monitor progress or deterioration of outcomes [15]. Lastly, measuring and reviewing PROs together with the patients could contribute to shared decision-making [16,17]. All these advantages contribute to a more patient-centered care approach.

Data generated within registries can also be used for research purposes. These real-world data are very valuable because they, among others, show us the community effectiveness [18]. Community effectiveness studies often show less benefit than efficacy studies or randomized controlled trials. Community effectiveness is determined by the following variables: efficacy, access (coverage), diagnostic certainty, upholding management guidelines by the treating physician, and patient compliance [18]. Furthermore, the organization and delivery of care may differ between medical specialists who treat IMIDs. For example, current rheumatoid arthritis guidelines do not have a laboratory monitoring program for tumor necrosis factor inhibitors, but the European psoriasis vulgaris guideline has one [19-21]. Another example is the use of therapeutic drug monitoring for tumor necrosis factor inhibitors in daily practice, which is more common in IBDs compared to rheumatic diseases [22,23]. The ability to use these data gives us the opportunity to better understand not only the differences between the organization and delivery of care, but also the differences or similarities in clinical outcomes as well as PROs between IMIDs. These data might help us improve the care of the individual patient through direct implementation of our research findings.

Finally, routine outcome measurements could be the first step toward the implementation of VBHC. VBHC aims to improve care by focusing on value instead of volume [24]. Value is defined as gained health divided by resources spent [24]. Gained health includes clinical outcomes as well as PROs and is therefore broader than we are currently used to [25,26]. As IMIDs are chronic conditions with fluctuating disease activity, longitudinal measurement of outcomes is necessary to evaluate the sustainability of health, incidence of complications, and side effects [25].

Implementation of longitudinal outcome measurement is currently upcoming. This is challenging because both health care providers and patients may find it time-consuming or impractical [27]. Therefore, there should be a practical way for physicians and patients to report and access these data, which could be through a web-based system. Electronic distribution of surveys before and during outpatient clinic visits has several benefits [28]: (1) patients can fill them out at home, (2) it streamlines the data-entry process, and (3) it allows for computer adaptive testing to reduce user burden. It also allows for easy data visualization and linkage with the electronic health record (EHR) for easy access by the health care providers.

The IMID registry was developed to implement digital routine outcome measurements in daily practice (1) to improve individual patient care, including shared decision-making, (2) for research purposes, and (3) as a first step toward the implementation of VBHC in patients with an IMID.

### Objectives of the IMID Registry

The objectives of the IMID registry include (1) improving patient care on an individual level through a patient-centered care approach, including shared decision-making; (2) collecting real-world data for research purposes, including evaluating community effectiveness; and (3) implementing VBHC for patients with IMIDs, for which—in our opinion—collection of patient- and provider-reported outcomes is a fundamental component.

## Methods

### Study Design

The IMID registry is a single-center prospective cohort within the Erasmus MC in Rotterdam, the Netherlands. Patients are recruited from the departments of rheumatology, immunology, gastroenterology, and dermatology. A steering committee was also formed and included representatives of all participating departments. All representatives were involved in the design of the registry and selection of outcome measures. The cohort combines provider-reported outcomes and PROs. On request, clinical and administrative data can be exported from the EHR and pharmacy database. These data may include disease activity measures, the number of outpatient clinic visits in a certain time period, laboratory results, and medication, including administration and dosage.

### Patients

All adult patients diagnosed with one of the following conditions are eligible: IA (rheumatoid arthritis, psoriatic arthritis, juvenile idiopathic arthritis, and spondyloarthritis), atopic dermatitis, psoriasis, IBD (Crohn disease and ulcerative colitis), uveitis, Behçet disease, sarcoidosis, or systemic vasculitis. Patients are excluded if they have insufficient knowledge of the Dutch language or are unable to fill out web-based questionnaires. Health care providers of each department ask eligible patients for informed consent, after which patients are included in their specific trajectory of the registry, which is based on the diagnosis that is made by the treating physician.

### Outcome Measures

For IBD and IA, the questionnaires were aligned with the corresponding International Consortium for Health Outcomes Measurement (ICHOM) outcome sets to make sure that all recommended questionnaires were included in the registry [29,30]. Generic and disease-specific outcomes that are used for each diagnosis include disease activity, disease damage as a result of active disease or complications (eg, changes in anatomy, physiology, pathology, or function), disease-specific and generic quality of life, treatment satisfaction, side effects, medication adherence, beliefs about medication, work productivity, disability, fatigue, and substance use. If available, validated questionnaires are used to measure these PROs. To reduce patient burden, the shortest versions of the questionnaires are used. See Table 1 for overarching outcomes and Table 2 for disease-specific outcomes. These outcomes can be linked to data from the EHR, such as medication, health care use, and disease duration.

**Table 1:** Generic outcomes and questionnaires.

Outcome measure		Used questionnaire	Time point				
			Baseline	3 months	6 months	Yearly	Indicated time (minutes)
**Treatment**							
	Side effects^a^		✓	✓	✓	✓	<0.5
	Beliefs about medication	BMQ^b^ necessity-concerns differential [31,32]	✓			✓	1.5
	Medication adherence	MARS-5^c^ [33]		✓	✓	✓	0.5
	Treatment satisfaction	VAS^d^ 0-100	✓	✓	✓	✓	0.5
**Disease impact**							
	Quality of life	EQ-5D-5L^e^ [34,35]	✓	✓		✓	1
	Quality of life	PROMIS-10^f^ [36,37]			✓	✓	1.5
	Quality of life	SF-36^g,h^ [38,39, 40]			✓	✓	4.5
	Fatigue	NRS^i^ 0-10	✓	✓	✓	✓	0.5
	Work disability	WPAI^j^ [41]	✓			✓	1
	Physical activity	iPAQ^k^ short form [42,43]^h^	✓	✓		✓	3
	Participation	Participation scale [44]^h^	✓	✓		✓	3.5

^a^Questionnaire reported by patients and physician.

^b^BMQ: Beliefs About Medicine Questionnaire.

^c^MARS-5: 5-item Medication Adherence Report Scale.

^d^VAS: visual analog scale.

^e^EQ-5D-5L: 5-level EQ-5D.

^f^PROMIS-10: 10-item Patient-Reported Outcomes Measurement Information System.

^g^SF-36: RAND 36-item short form survey.

^h^These questionnaires were used earlier, but are not part of the questionnaire set anymore.

^i^NRS: numeric rating scale.

^j^WPAI: Work Productivity and Activity Impairment Questionnaire.

^k^iPAQ: International Physical Activity Questionnaire.

**Table 2:** Disease-specific outcomes and questionnaires.

Disease group, disease, and questionnaires used		Outcome measure				
		Disease activity	Damage	Quality of life	Other^a^	Indicated time (minutes)
**Inflammatory** **arthritis**						
	DAS^b^-28^c^ [45]	✓				
	Disease damage^c^		✓			<0.5
	HAQ-DI^d^ [46,47]				✓	2.5
	DMARD^e^ side effects^c^				✓	<0.5
	BASDAI^c,f^ (spondyloarthritis) [48,49]	✓				1
**Inflammatory bowel disease**						
	IBD^g^-control-8 [50,51]	✓				1
	iMTA^h^ PCQ^i^ [52]			✓		1
	Manitoba IBD Index [53]	✓				0.5
	Montreal classification^c,j^ [54]			✓		0.5
	Disease damage^j^		✓			<0.5
	IBDQ^k^ [55]^j^			✓		5.5
	Harvey Bradshaw Index (Crohn disease) [56]^j^	✓				0.5
**Immunology**						
	BDCAF^l,m^ (Behçet disease) [57]	✓				2.5
	Sarcoidosis disease activity^c^ (sarcoidosis)	✓				0.5
	VFQ^n^-25 (uveitis) [58]			✓		10
	BVAS^c,o^ (vasculitis) [59]	✓				0.5
	VDI^c,p^ (vasculitis) [60]		✓			0.5
**Dermatology**						
	ADCT^q^ [61]	✓				0.5
	NRS^r^ disease burden^c^		✓			0.5
	DLQI^s^ [62]		✓			1.5
	PGA^t^ or IGA^u^ scale^c^	✓				<0.5
	PASI^c,v^ (psoriasis) [63]	✓				0.5
	POEM^w^ (atopic dermatitis) [64]	✓				1
	EASI^x^ (atopic dermatitis) [65]	✓				1

^a^Questionnaires involving side effects, physical functionality, and productivity loss.

^b^DAS: Disease Activity Score.

^c^Questionnaire reported by physician only.

^d^HAQ-DI: Health Assessment Questionnaire Disability Index.

^e^DMARD: disease-modifying antirheumatic drug.

^f^BASDAI: Bath Ankylosing Spondylitis Disease Activity Index.

^g^IBD: inflammatory bowel disease.

^h^iMTA: Institute for Medical Technology Assessment.

^i^PCQ: Productivity Cost Questionnaire.

^j^These questionnaires were asked earlier, but are not part of the questionnaire set anymore.

^k^IBDQ: Inflammatory Bowel Disease Questionnaire.

^l^BDCAF: Behçet Disease Current Activity Form.

^m^Questionnaire reported by patients and physician.

^n^VFQ: Visual Function Questionnaire.

^o^BVAS: Birmingham Vasculitis Activity Score.

^p^VDI: Vasculitis Damage Index.

^q^ADCT: Atopic Dermatitis Control Tool.

^r^NRS: numeric rating scale.

^s^DLQI: Dermatology Life Quality Index.

^t^PGA: patient global assessment.

^u^IGA: Investigator’s Global Assessment.

^v^PASI: Psoriasis Area and Severity Index.

^w^POEM: Patient-Oriented Eczema Measure.

^x^EASI: Eczema Area and Severity Index.

### Generic Outcomes

#### Patient- and Physician-Reported Side Effects

Because health care providers might underreport side effects, both patients and physicians are asked about the occurrence of side effects [66]. If any side effects occur, respondents are asked to clarify the time of onset, severity, and if there are any persisting side effects after medication dosage was changed or stopped. The physician is asked to clarify if medication is switched or if additional medication (eg, antibiotics) is needed.

#### Beliefs About Medication

The Beliefs About Medicine Questionnaire has been validated for assessing medication beliefs in patients with somatic chronic illnesses and consists of a specific and general section [31,32]. For this cohort, we only use the “specific” section that measures patient beliefs about the necessity of prescribed medication to treat their disease and their concerns about potential adverse events of taking the medication. All questions are rated on a 5-point Likert scale. Higher scores indicate stronger beliefs about necessity of their medication or concerns.

#### Medication Adherence

The 5-item Medication Adherence Report Scale is used to assess nonadherence to medication using a 5-point Likert scale [33]. It addresses nonadherence in a nonjudgmental way, encourages patients to answer truthfully and avoid socially desirable answers. Higher scores indicate higher levels of self-reported adherence.

#### Treatment Satisfaction

Patients are asked to indicate their feeling of satisfaction with the effect of their current treatment on disease activity on a visual analog scale (VAS) from 0 to 100. Higher scores indicate higher treatment satisfaction.

#### Fatigue

This questionnaire asks patients to rate their fatigue level in the last 7 days using a numeric rating scale of 1 to 10. A higher score corresponds with more fatigue experienced.

#### (Health-Related) Quality of Life

(Health-related) quality of life is measured with 2 different questionnaires: the 5-level EQ-5D and the Patient-Reported Outcomes Measurement Information System (PROMIS-10) [34-37]. The 5-level EQ-5D is used to measure utility scores based on the Dutch reference values and consists of 5 questions representing 5 dimensions of health, namely, mobility, self-care, daily activities, pain and discomfort, and anxiety and depression [34,35,67]. Each dimension can be scored on a 5-point Likert scale. Higher scores indicate more reported problems in the stated health dimension. The sixth question consists of a VAS general health.

The PROMIS-10 measures general health-related quality of life using 10 questions on physical, mental, and social health, pain, fatigue, and overall perceived quality of life [36,37]. Answers are given using a 5-point Likert scale or a VAS from 0 to 10. Outcomes are summarized into a summary score for global physical health and global mental health with accompanying *t* scores that compare the patients’ health to the general population.

#### Work Productivity

The Work Productivity and Activity Impairment questionnaire [41] measures sick leave or unemployment (absenteeism) and work impairment due to the disease (presenteeism) during the past 7 days. The questionnaire contains 6 questions regarding the effect of disease on work and productivity.

#### Other Questionnaires

Over the years, some of the used questionnaires have changed or have been adapted based on feedback from health care providers and patients. These questionnaires are shown in Tables 1 and 2.

### Disease-Specific Outcome Measures

For each disease (group), disease-specific outcome measures were chosen. They cover outcomes about disease activity and damage, quality of life, functional ability, side effects, and productivity loss. These outcome measures along with their accompanying questionnaires are shown in Table 2.

### Patient and Public Involvement

Patients were not involved in the design of the registry, but our patient participation panel and included IMID registry patients will be regularly asked to evaluate the questionnaire system, including the number and relevance of the questionnaires. The registry will be optimized based on their feedback.

### Data Collection

To limit user burden, the questionnaires are divided over different time points. At baseline and 2 weeks before the outpatient clinic visits, an invitation to fill out new questionnaires is sent to the patient by email. The health care providers fill out the surveys during or right after the consultation. After 1 year, the questionnaires are filled out yearly. However, an exception is made for patients with IA, IBD, and atopic dermatitis, because they are asked to fill out questionnaires before each outpatient clinic visit or at fixed intervals, which were added on request of the participating departments. Surveys are filled out using a web-based questionnaire form (LimeSurvey) [68]. The questionnaires are managed by a data capture system (GemsTracker) [69], which is integrated in the EHR.

### Data Visualization

Data are accessible through a web-based form, which is linked to the EHR. PRO scores are shown in the same manner as, for example, laboratory results and are also directly accessible through the EHR and visualized in graphs over time. For IA and atopic dermatitis, a dashboard has been developed, which allows visualization of several outcome measures over time in a comprehensive manner.

### Training and Monitoring of the Implementation Process

Group trainings were given at the participating departments on the usage of the registry and how to access the data from the EHR. During the implementation phase, health care providers were supported during their consultations on the use of the data and how to discuss and interpret the PRO outcomes with the patients, including shared decision-making. Every 3 months, the IMID steering group comes together to discuss the progress of the registry, and every month multidisciplinary (scientific) meetings are held for all participating health care providers.

### Ethics Approval

The IMID registry is a quality of care improvement initiative. Therefore, written informed consent is not mandatory for this purpose. Patients are asked by their health care provider to participate and give oral informed consent. In the web-based questionnaire form, they can opt-in for the use of their pseudonymized data for future scientific research. The Medical Ethics Committee of the Erasmus MC deemed the study to not be subject to Dutch law (WMO; Medical Research Involving Human Subjects Act) and provided a waiver (MEC-2018-1075).

## Results

The IMID registry is an ongoing cohort with no end date. Inclusion started in April 2018. Until September 2022, a total of 1417 patients have been included in the registry. The included patients belong to the dermatology (n=477, 34%), immunology (n=153, 11%), gastroenterology (n=327, 23%), and rheumatology (n=460, 32%) departments.

The mean age at inclusion was 46 (SD 16) years, and 56% of the included patients are female. In Table 3, the demographics and baseline response rates are given, stratified for medical specialty. A total of 84% of patients filled out their questionnaires at baseline, which declined to 72% after 1 year of follow-up. In addition, a total of 92% of patients gave written informed consent for usage of their pseudonymized data for research purposes.

**Table 3:** Demographics and baseline response rate stratified for medical specialty.

Medical specialty	Response rate,^a^ n (%)	Age (years), mean (95% CI)	Sex (female), %
Dermatology^b^ (N=477)	378 (79)	41 (39-42)	43
Immunology^c^ (N=153)	126 (82)	50 (47-52)	58
Gastroenterology^d^ (N=327)	314 (96)	46 (44-47)	53
Rheumatology^e^ (N=460)	415 (90)	49 (47-51)	68

^a^Percentage of patients who filled in the patient satisfaction questionnaire at baseline.

^b^Atopic dermatitis, psoriasis, and unclassified dermatologic diseases.

^c^Uveitis, Behçet disease, sarcoidosis, and systemic vasculitis.

^d^Crohn disease and ulcerative colitis.

^e^Rheumatoid arthritis, psoriatic arthritis, juvenile idiopathic arthritis, and spondyloarthritis.

## Discussion

The IMID registry is a web-based system linked to the EHR that collects provider-reported outcomes and PROs. These outcomes are not only used to improve care for the individual patient with IMIDs including facilitation of shared decision-making, but they are also used for research purposes. Moreover, routine outcome measurements are an essential component in the implementation of VBHC. The registry has been used by the departments of rheumatology, gastroenterology, dermatology, and immunology, since 2018. Over the years, the outcome sets have been improved and adapted based on feedback from health care providers, patients, and current scientific insights. During the first 4 years, more than 1400 patients were included, which represents a broad uptake from all participating departments.

Previous publications have shown that patients with IMIDs may cope with lower quality of life, more disability, increased fatigue, pain, and productivity loss, but they also have more psychiatric comorbidity and medication-related adverse events [1,10-13,70]. The questionnaires were chosen to cover all aforementioned topics in a comprehensive manner to best reflect our overall health status of patients with IMIDs, but we also included the domains that matter most to our patients.

We chose to use generic questionnaires for several types of IMIDs, since these diseases may share common problems and patients may suffer from overlapping conditions within the IMID spectrum [1]. These diseases also share treatment strategies that may cause similar adverse events [1-9,13]. Generic questionnaires about these topics may lead to (1) evaluation of outcomes across patients with different conditions, (2) feedback from a larger patient-panel, and (3) improvements through the steering group’s broad perspective.

Although the IMID registry has improved over the years, the cohort has some limitations. First, patients did not participate in the design of this registry. Currently, the IMID registry has been evaluated by 42% (n=467) of the patients that filled out a questionnaire in the past year. The survey included questions on the use of the system and length and content of the questionnaires. They also provided feedback on which topics they would like to discuss during consultations. The registry was adjusted based on their feedback. A couple of examples are rephrasing of some questions to make them more understandable, lowering the number of questionnaires before each visit, and changing the user interface to make them accessible for phone or tablet users. These evaluation rounds will be planned regularly.

The generalizability of the registry might be limited due to selection bias. Not all treating physicians use the registry during their outpatient clinic visits, and also not all patients are included or fill out their questionnaires. This could be due to the fact that patients cannot read or understand Dutch, have problems using a digital system, or the fact that the PROs are not discussed during consultation. Moreover, only patients who need specialized care are treated at the Erasmus MC. Finally, patients who tend to avoid care may cause nonresponse or selection bias. Although most of the included patients filled out their questionnaires at baseline, this percentage declines during follow-up. This is partly due to patients not filling out the questionnaires, but also on occasion, the questionnaire were not sent to the patient before the follow-up visit. In the near future, we hope to address these limitations by automatic inclusion of patients, giving them the opportunity to fill out questionnaires in the waiting room as well as by providing validated questionnaires in different languages. Providing more information about the use of the data, automatic distribution of questionnaires, as well as providing feedback about the filled out questionnaires during every consultation could also increase the response rate during follow-up.

We will also keep improving the IMID registry to optimize its relevance and feasibility. Currently, data are collected and visualized using a separate data collection tool, which is linked to the EHR. In the future, we will incorporate data collection directly into the EHR, as well as making it possible for the patient to visualize the data in a mobile app. Although routine outcome measurements are—in our opinion—a fundamental component in the implementation of VBHC, we hope to incorporate more VBHC principles in the future, as well as collaborating with other hospitals in the region, which has already been established for IBD and will soon be established for rheumatology [71].

In conclusion, the IMID registry provides a web-based outcome measurement system, which could facilitate the principles of VBHC, improve care on an individual patient level, and provide real-world data for research purposes.

## Abbreviations

EHR: electronic health record
IA: inflammatory arthritis
IBD: inflammatory bowel disease
IMID: immune-mediated inflammatory disease
PRO: patient-reported outcome
PROMIS-10: Patient-Reported Outcomes Measurement Information System
VAS: visual analogue scale
VBHC: value-based health care

## Data Availability

Data are available from the corresponding author on reasonable request.
